# High-Resolution Azimuth Estimation Method Based on a Pressure-Gradient MEMS Vector Hydrophone

**DOI:** 10.3390/mi17020167

**Published:** 2026-01-27

**Authors:** Xiao Chen, Ying Zhang, Yujie Chen

**Affiliations:** 1 Information Science and Technology College, Dalian Maritime University, Dalian 116026, China; chenxiao620@dlmu.edu.cn; 2 Department of Electronic Engineering, Ocean University of China, Qingdao 266100, China; zhying_note@163.com; 3 Qian Xuesen College, Qingdao Huanghai University, Qingdao 266100, China

**Keywords:** pressure-gradient MEMS vector hydrophone, azimuth estimation, high-resolution, improved particle swarm optimization (IPSO), underwater acoustic sensor

## Abstract

The pressure-gradient Micro-Electro-Mechanical Systems (MEMS) vector hydrophone is a novel type of sensor capable of simultaneously acquiring both scalar and vectorial information within an acoustic field. Conventional azimuth estimation methods struggle to achieve high-resolution localization using a single pressure-gradient MEMS vector hydrophone. In practical marine environments, the multiple signal classification (MUSIC) algorithm is hampered by significant resolution performance loss. Similarly, the complex acoustic intensity (CAI) method is constrained by a high-resolution threshold for multiple targets, often resulting in inaccurate azimuth estimates. Therefore, a cross-spectral model between the acoustic pressure and the particle velocity for the pressure-gradient MEMS vector hydrophone was established. Integrated with an improved particle swarm optimization (IPSO) algorithm, a high-resolution azimuth estimation method utilizing this hydrophone is proposed. Furthermore, the corresponding Cramér-Rao Bound is derived. Simulation results demonstrate that the proposed algorithm accurately resolves two targets separated by only 5° at a low signal-to-noise ratio (SNR) of 5 dB, boasting a root mean square error of approximately 0.35° and a 100% success rate. Compared with the CAI method and the MUSIC algorithm, the proposed method achieves a lower resolution threshold and higher estimation accuracy, alongside low computational complexity that enables efficient real-time processing. Field tests in an actual seawater environment validate the algorithm’s high-resolution performance as predicted by simulations, thus confirming its practical efficacy. The proposed algorithm addresses key limitations in underwater detection by enhancing system robustness and offering high-resolution azimuth estimation. This capability holds promise for extending to multi-target scenarios in complex marine settings.

## 1. Introduction

Acoustic waves are the primary medium for long-distance underwater information exchange. Conventional hydrophones capture only the scalar pressure component of a sound field [1], whereas a complete characterization requires vector quantities such as particle velocity and the pressure gradient. Vector hydrophones fulfill this requirement by simultaneously measuring acoustic pressure and particle velocity. Thus, they offer more complete spatial information and exhibit frequency-independent directivity [2]. This unique advantage, stemming from the co-located measurement of complementary field components, allows a single vector hydrophone to estimate a target’s direction-of-arrival (DOA) [3,4]. Accurate, high-resolution azimuth estimation of underwater acoustic targets is of pivotal importance. Refining such techniques holds theoretical and practical value for the entire domain of underwater acoustic detection.

A vector hydrophone typically integrates acoustic pressure sensor or particle velocity sensor, adhering to the co-located principle where the geometric, mass, and phase centers coincide [5]. Based on operational principles, vector hydrophones are primarily categorized into two categories: pressure-gradient and co-vibrating designs. Compared to co-vibrating vector hydrophones, the pressure-gradient type has distinct practical advantages. It features a simpler mechanical structure. Its deployment is easier. It is also less sensitive to mechanical vibration. These characteristics make it highly suitable for diverse underwater applications [6]. The integration of MEMS technology enables hydrophone miniaturization while conferring advantages such as high sensitivity, excellent unit-to-unit consistency, and ease of integration [7]. Specifically, the pressure-gradient MEMS vector hydrophone estimates the pressure gradient by measuring the pressure difference between two closely spaced points in the acoustic field, which is then translated into particle velocity vector information. Consequently, it is capable of target azimuth estimation and exhibits inherent resistance to acceleration-induced noise. These attributes confer significant advantages for deployment on mobile platforms like Unmanned Underwater Vehicles (UUVs) and Autonomous Underwater Vehicles (AUVs) [8].

The pressure-gradient vector hydrophone operates on the principle of finite-difference to directly exploit its vector sensing capability for signal processing. Consequently, it can be equivalently treated as a circular array of acoustic pressure sensors. This equivalency allows flexible adaptation of various array-based signal processing methodologies. For DOA estimation with a single vector hydrophone, the MUSIC algorithm is a seminal high-resolution method [9]. It operates by constructing a spatial spectrum that exploits the orthogonality between the signal and noise subspaces, where the peak indicates the estimated azimuth. In contrast, the CAI method represents a classical, physically intuitive approach that directly estimates the azimuth from the co-located pressure and particle velocity channels [10]. It differs fundamentally from MUSIC as it requires no eigen-decomposition or spectral peak search. Instead, the target azimuth is derived directly from the cross-spectral density between the co-located acoustic pressure and particle velocity measurements. Lim et al. [11] investigated a MUSIC-like algorithm by incorporating different noise subspace models, which yielded improved spatial spectral peak detection in complex underwater environments. The MUSIC algorithm, while capable of handling multiple targets, suffers from performance degradation at low SNR due to subspace leakage, which generates spurious peaks in the azimuth spectrum and impairs resolution. Moreover, its high computational burden is at odds with real-time, high-resolution processing requirements. Although MUSIC-like variants offer improved accuracy in complex noise environments, their even higher algorithmic complexity further precludes real-time application. Conversely, the CAI method imposes a relatively high minimum separation requirement for resolving multiple targets, which constrains its high-resolution performance. These limitations constitute the primary impetus for developing new high-resolution azimuth estimation algorithms [12,13].

This paper tackles the limited azimuth resolution of single vector hydrophones by proposing a high-resolution algorithm based on a pressure-gradient MEMS sensor. The method constructs a highly discriminative objective function from the pressure-velocity cross-spectrum and employs an IPSO algorithm with adaptive learning for efficient global optimization. This strategy leverages inherent vector-field correlations and overcomes the accuracy-efficiency trade-offs of traditional search methods. The algorithm delivers superior resolution and accuracy in low-SNR and multi-target conditions, suppresses common-mode noise, and has been validated at sea. Although parameter-dependent and requiring further testing in extreme multipath environments, it offers lower computational complexity suitable for real-time embedded systems. Simulations and field data confirm its outperformance over CAI and MUSIC in both resolution and robustness, establishing a valuable new framework for high-precision azimuth estimation with clear applications in underwater tracking.

## 2. Materials and Methods

### 2.1. MEMS Acoustic Pressure Sensor

**Figure 1:**
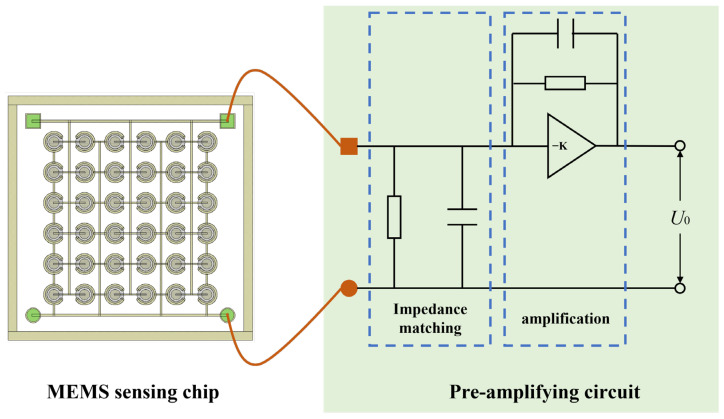
Structure of the MEMS acoustic pressure sensor. The MEMS sensing chip is wire-bonded to the preamplifier circuit.

**Figure 2:**
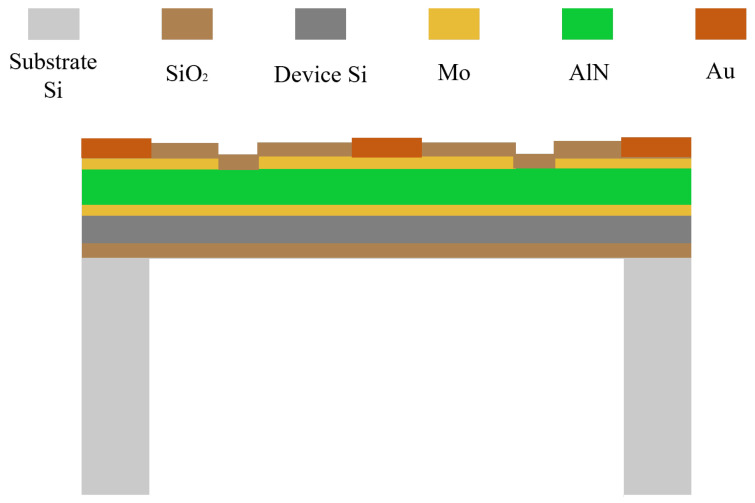
Cross-sectional schematic of the MEMS sensing unit cell.

The MEMS acoustic pressure sensor primarily comprises a MEMS sensing chip and a conditioning amplifier circuit, as illustrated in Figure 1. In operation, the piezoelectric film within the MEMS sensing chip deforms under acoustic pressure, inducing proportional charges of opposite polarity on its surfaces. This directly transduces the acoustic signal into a weak electrical output [14]. When AlN serves as the piezoelectric layer, its theoretical receiving sensitivity is approximately an order of magnitude higher than that of PZT [15]. The structure of the MEMS sensing unit is shown in Figure 2. Fabricated on a silicon-on-insulator (SOI) substrate, it consists of a device layer, a buried oxide layer, and a handle layer. The core sensing element is a Mo-AlN-Mo sandwiched structure. To enhance acoustic collection efficiency within a constrained footprint, the chip implements a 6 × 6 planar array of these units. This design effectively increases the equivalent sensitive area, thereby improving overall sensitivity and noise immunity. The electrical signal from the MEMS sensing chip is extremely weak and prone to noise interference. To address this, a dedicated conditioning circuit with low-noise performance and high input impedance was designed. To minimize parasitic capacitance and lead interference, the circuit is placed adjacent to the sensing chip. This ensures that the weak signal is amplified faithfully without significant degradation.

**Figure 3:**
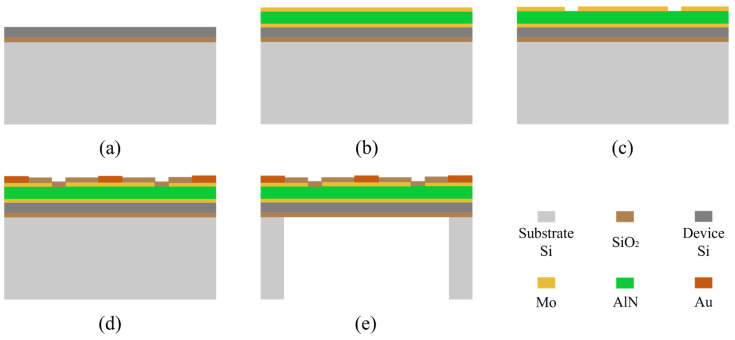
Fabrication process flow of the MEMS sensing chip. (**a**) SOI wafer cleaning. (**b**) Sputtering of the Mo-AlN-Mo piezoelectric stack. (**c**) Electrode patterning via photolithography and etching. (**d**) Deposition and lift-off of the Au bonding pads. (**e**) Back-side cavity etching by DRIE.

As shown in Figure 3, the fabrication process commenced with a 6-inch SOI wafer, comprising a 5-μm device layer, a 1-μm buried oxide layer, and a 400-μm handle layer. The wafer surface was thoroughly cleaned prior to processing. A 20 nm-thick seed layer was first deposited by sputtering. Subsequently, a tri-layer Mo-AlN-Mo piezoelectric stack with thicknesses of 0.3 μm, 2 μm, and 0.3 μm, respectively, was sputtered. A series of photolithography and etching steps were then performed on the deposited films to pattern the electrodes into annular structures. The inner ring, with a radius equal to 70% of the vibrating diaphragm radius, functions as the top electrode. Conversely, the outer ring, with a width constituting 10% of the diaphragm radius, acts as the bottom electrode. Next, titanium (Ti) and gold (Au) layers were deposited and lifted off to form bonding pads for subsequent wire interconnection. Finally, to release the vibrating diaphragm, deep reactive-ion etching (DRIE) was employed to etch through the silicon handle layer from the backside, creating a backside cavity with a radius of 200 μm.

**Figure 4:**
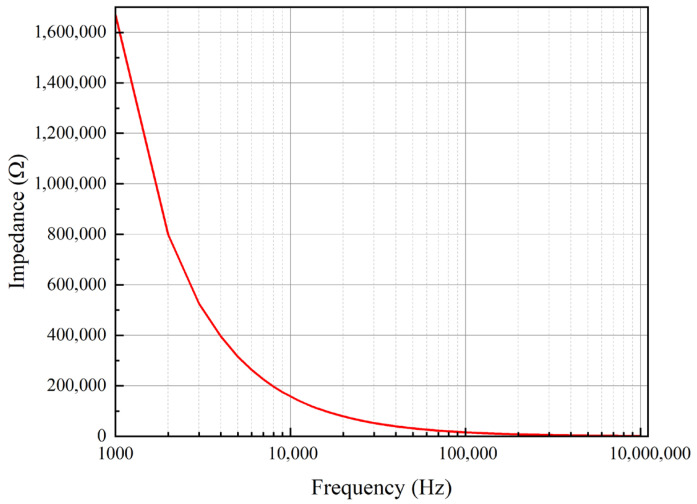
Impedance characterization of the MEMS sensing chip.

**Figure 5:**
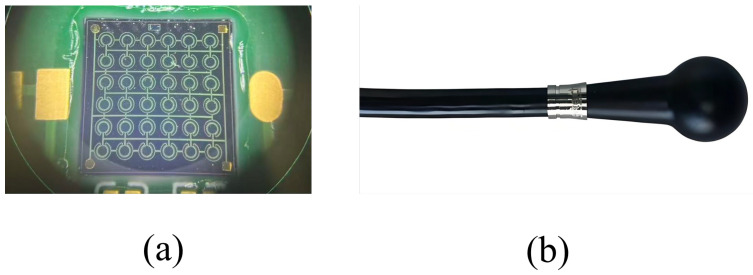
MEMS acoustic pressure sensor. (**a**) Assembly of the sensing chip on the preamplifier board. (**b**) Packaged sensor device.

The MEMS sensing chip outputs a weak electrical charge signal, which necessitates a preamplifier circuit for signal amplification to enable effective measurement. A gain of 40 dB was designed for this purpose. Furthermore, as MEMS sensing chips are inherently high-impedance devices, the preamplifier circuit must also provide appropriate impedance matching. The impedance of the MEMS sensing chip was characterized using a semiconductor parameter analyzer (4200A-SCS, Keithley Instruments, LLC, Cleveland, OH, USA), with the test setup illustrated in Figure 4. Measurements at 1 kHz yielded an impedance of 1.67 MΩ for the sensing chip. To minimize signal loading, the input impedance of the preamplifier was designed to be over two orders of magnitude higher. Therefore, the preamplifier circuit serves the dual function of impedance matching and signal amplification. A photograph of the MEMS sensing chip assembled onto the preamplifier circuit board is presented in Figure 5a.

Polyurethane was selected to encapsulate the MEMS acoustic pressure sensor, owing to its acoustic impedance, which closely matches that of water, and its low acoustic attenuation coefficient. This material provides excellent acoustic transparency, water resistance, and seawater corrosion resistance [16]. The packaged sensor, shown in Figure 5b, features a spherical housing with a diameter of 22 mm.

### 2.2. Design of the Pressure-Gradient MEMS Vector Hydrophone

**Figure 6:**
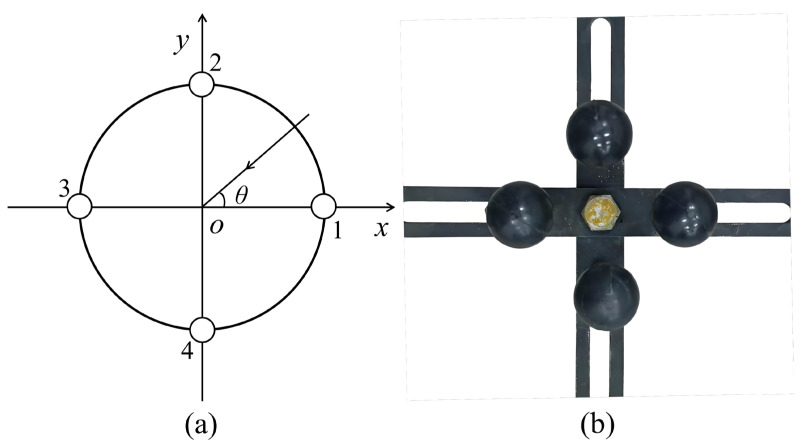
Two-dimensional pressure-gradient MEMS vector hydrophone. (**a**) Schematic of the working principle. (**b**) Photograph of the physical assembly.

The pressure-gradient MEMS vector hydrophone operates by approximating the acoustic pressure gradient using a spatial finite-difference scheme. This gradient is then related to medium particle acceleration via Euler’s equation, ultimately yielding the particle velocity vector. The hydrophone is implemented using orthogonal dipole pairs. Specifically, the two-dimensional design presented here comprises two such pairs, formed by four matched MEMS acoustic pressure sensors [17]. As shown in Figure 6a, elements 1–4 represent these sensors. The distance between opposing elements (e.g., 1 and 3 or 2 and 4) is 2*r*, and *θ* denotes the target’s azimuth angle. Figure 6b shows the physical realization, with the sensors secured in a precision-machined cross-shaped steel fixture.

Given that the sound source is in the far field with a negligible elevation angle, the problem is considered in the two-dimensional horizontal plane. The pressure-gradient MEMS vector hydrophone operates from 20 Hz to 3000 Hz, with a dipole spacing (2*r*) of 0.13 m. This spacing is significantly smaller than the wavelength (*λ*) at the band’s center frequency (2*r* << *λ*). Therefore, the average voltage output from the four MEMS acoustic pressure sensors accurately represents the acoustic pressure at the hydrophone’s center point.

(1) $$U_{p}(t)=\frac{1}{4}\left(U_{1}(t)+U_{2}(t)+U_{3}(t)+U_{4}(t)\right)$$

where *U_p_*(*t*) represents the instantaneous acoustic pressure at the center, and *U_i_* (*i* = 1, 2, 3, 4) are the respective voltage outputs of the four MEMS acoustic pressure sensors constituting the received signal.

Based on the finite-difference method, this can be approximated by

(2) $$\nabla U_{x}\approx \frac{\Delta U}{\Delta x}=\frac{U_{1}(t)-U_{3}(t)}{2r}$$

(3) $$\nabla U_{y}\approx \frac{\Delta U}{\Delta y}=\frac{U_{2}(t)-U_{4}(t)}{2r}$$

where $\nabla U_{x}$ and $\nabla U_{y}$ represent the pressure-gradient. Therefore, according to Euler’s formula, the particle velocity components along the *x* and *y* axes can be derived as [6]

(4) $$v_{x}(t)\approx -\frac{1}{\rho }\int \nabla U_{x}dt$$

(5) $$v_{y}(t)\approx -\frac{1}{\rho }\int \nabla U_{y}dt$$

where *ρ* is the density of the medium. From this relationship, the output of the pressure-gradient MEMS vector hydrophone can be concisely represented by

(6) $$\left\{\begin{array}{l} U_{0}(t)=U_{p}(t)\\ v_{x}(t)=-\frac{1}{\rho }\int \nabla Udt\\ v_{y}(t)=-\frac{1}{\rho }\int \nabla Udt \end{array}\right..$$

A unique capability of the pressure-gradient MEMS vector hydrophone is the synchronous, co-located measurement of the acoustic pressure *U*_0_ and the orthogonal particle velocity components ***v**_x_* and ***v**_y_*. Among them, the vibration velocity information contains the target orientation information. Consequently, by jointly processing the acoustic pressure and particle velocity, the azimuth of an underwater target can be estimated.

**Figure 7:**
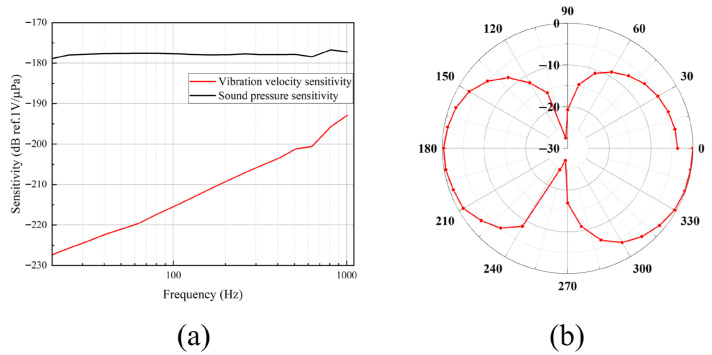
Calibration results of the two-dimensional pressure-gradient MEMS vector hydrophone. (**a**) Sensitivity. (**b**) Directivity.

The pressure-gradient MEMS vector hydrophone was calibrated in a standing-wave tube. As shown in Figure 7a, the measured pressure sensitivity level is –178 dB (re 1 V/μPa), and the particle velocity sensitivity at 630 Hz is –201.2 dB (re 1 V/μPa). The measured vibration velocity sensitivity decays at 6 dB/octave from 20 Hz to 1000 Hz, consistent with the theoretical frequency response model. Its directivity pattern, presented in Figure 7b, exhibits the classic “figure-of-eight” shape. The achieved null depth of over 20 dB validates that the vector channel resolution meets the design target.

### 2.3. Improved Particle Swarm Optimization Algorithm

The target azimuth angle is intrinsically linked to the cross-spectra between the acoustic pressure and particle velocity measured by the pressure-gradient MEMS vector hydrophone. This relationship is derived by first applying the Fourier transform to the pressure channel output *U*_0_ and the orthogonal velocity components ***v**_x_* and ***v**_y_*, yielding *U*_0_(*ω*), ***v**_x_*(*ω*), and ***v**_y_*(*ω*). The required pressure-velocity cross-spectra are then given by [10]:

(7) $$S_{i}(\omega )=U_{0}(\omega )\cdot v_{i}^{*}(\omega )(i=x,y)$$

where *ω* is the frequency and the superscript * denotes the complex conjugate.

The particle velocity and acoustic pressure are in-phase signals. A fundamental property of the Fourier transforms dictates that the energy of two in-phase inputs concentrates in the real part of their cross-spectrum. It follows that the target signal energy is largely contained within the real component of the mutual spectral output, with the imaginary component consisting mainly of interference energy. Therefore, the final, usable cross-spectrum is defined as the real part:

(8) Ii(ω)=ReU0(ω)⋅vi∗(ω)(i=x,y).

Thus, the azimuth estimation problem is formulated as an unconstrained optimization. The objective function is defined as the mean square error (MSE) between the measured and theoretical pressure-velocity cross-spectra, where a smaller MSE corresponds to a more accurate estimate. The theoretical cross-spectrum, which is a function of the azimuth *θ*, is constructed as:

(9) $${\hat{I}}_{x}(\theta )=\cos(\theta )$$

(10) $${\hat{I}}_{y}(\theta )=\sin(\theta ).$$

The objective function can be expressed as [18]

(11) $$J(\theta )=\frac{1}{N^{2}}\sum_{k=1}^{N}\left[{\left(I_{x}(k)-{\hat{I}}_{x}(\theta )\right)}^{2}+(I_{y}(k)-{\hat{I}}_{y}{\left(\theta \right)}^{2}\right]$$

where *N* denotes the total number of effective frequency bins within the target band, *k* = 1, 2, …, *N* indexes these bins, and *θ* is the azimuth angle to be estimated.

The IPSO algorithm is employed to search for the angle *θ* that minimizes the objective function, which is thus identified as the optimal azimuth estimate. In IPSO, each candidate solution is a “particle.” The algorithm’s efficiency stems from particles possessing both individual and social learning capabilities, enabling rapid convergence to the optimum within few iterations [18].

Within the IPSO framework, each particle is characterized by its current position and velocity [19]. These two state variables evolve according to the following iterative update rules, which balance individual memory with social learning:

(12) $$v_{i,d}(t+1)=wv_{i,d}(t)+\eta_{1}(p_{i,d}(t)-z_{i,d}(t))+\eta_{2}(p_{g,d}(t)-z_{i,d}(t))$$

(13) $$z_{i,d}(t+1)=z_{i,d}(t)+v_{i,d}(t+1)$$

where *z_i_*_,_ *_d_* (*t* + 1) represents the updated position, and *v_i, d_* (*t* + 1) the updated velocity, for particle *i* in dimension *d*. The term *w* is the inertia weight. The parameters *η*_1_ (individual) and *η*_2_ (social) are acceleration coefficients that scale the influence of the particle’s personal best *p_i, d_* (*t*) and the swarm’s global best *p_g, d_* (*t*). A standard stability criterion requires the total acceleration *η* = *η*_1_ + *η*_2_ ≤ 4 to prevent divergent behavior. Consequently, the values *η*_1_ = 2.5 and *η*_2_ = 1.5 are chosen for our implementation.

**Figure 8:**
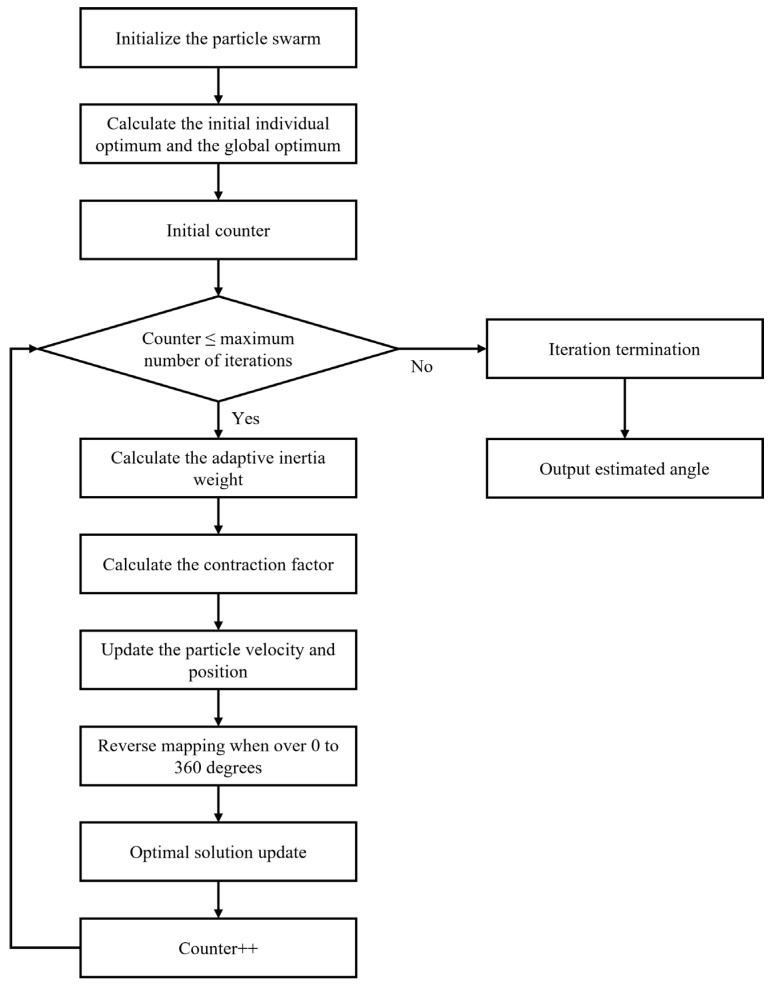
Flowchart of the IPSO algorithm for azimuth estimation.

The workflow of the IPSO algorithm for azimuth estimation is outlined in Figure 8. The swarm size and number of iterations are optimized by evaluating the algorithm’s performance under controlled conditions. Based on a quantitative assessment that prioritized both estimation precision and computational efficiency, the final configuration uses 20 particles and 30 iterations. The swarm is initialized with 20 particles. Each particle is assigned a random position uniformly distributed between 0° and 360° and zero initial velocity. Each particle’s personal best is set to its initial position, while the global best is assigned to the position corresponding to the minimum initial objective function value. Iterative optimization then commences, updating velocities and positions each generation. An adaptive inertia weight is employed to balance global exploration and local exploitation. This weight decreases linearly over iterations. A higher initial value promotes a broad search, which helps avoid local optima. As it subsequently reduces, the search focus narrows, facilitating refined convergence near the optimum. The adaptive inertia weight makes the improved algorithm significantly less sensitive to the specific settings for the number of particles and iterations. This provides strong fault tolerance for these parameters, enhancing the algorithm’s robustness in practical applications.

(14) $$w=w_{s}-(w_{s}-w_{e})\cdot \left(C/M\right)$$

where *w_s_* = 0.9 and *w_e_* = 0.2 are the initial and final inertia weights, respectively; *C* is the current iteration index; and *M* = 30 is the maximum number of iterations. Additionally, a constriction factor is applied to the velocity update to prevent divergence and enhance convergence stability [19].

(15) $$s=\frac{2}{\left|2-\eta -\sqrt{\eta^{2}-4\eta }\right|}$$

where *s* denotes the constriction factor. After updating the inertia weight and applying the constriction factor, the particle velocities and positions are recalculated, and the personal and global best solutions are updated accordingly. The algorithm terminates after reaching the maximum iterations, and the global best position is output as the final optimal azimuth estimate.

## 3. Results and Discussion

### 3.1. Performance Metrics

The accuracy of the positioning algorithm is quantified by the Root Mean Square Error (RMSE) [20,21].

(16) $$RMSE=\sqrt{\frac{1}{CS\times K}\sum_{n=1}^{CS}\sum_{k=1}^{K}{\left(\theta_{k}-{\hat{\theta }}_{n,k}\right)}^{2}}$$

where *K* is the number of sound sources, *CS* denotes the number of independent Monte Carlo trials, and *θ_k_* is the true azimuth of the *k*-th source. Here, ${\hat{\theta }}_{n,k}$ represents its estimated azimuth in the *n*-th trial.

The algorithm’s performance is evaluated by the successful resolution rate, defined as the percentage of Monte Carlo trials in which the target is correctly resolved.

(17) $$\kappa =\frac{L_{p}}{L_{all}}\times 100\%$$

where *L_p_* is the number of trials where the RMSE of the DOA estimate is ≤ 2°, and *L_all_* is the total number of trials.

### 3.2. Cramér-Rao Bound

The Cramér-Rao Bound (CRB) serves as the principal criterion for evaluating the precision of unbiased estimators, establishing the lowest possible variance attainable by such estimators [22]. In the context of the pressure-gradient MEMS vector hydrophone, the CRB specifies the fundamental lower limit for azimuth estimation variance by characterizing the Fisher information content of the received acoustic data.

Under the narrowband far-field assumption, the pressure-gradient MEMS vector hydrophone array is characterized by its steering vector ***a***(*θ*, *r*) [6].

(18) $$a(\theta ,r)=\left[\begin{array}{c} a_{p}(\theta ,r)\\ a_{x}(\theta ,r)\\ a_{y}(\theta ,r) \end{array}\right]=\left[\begin{array}{c} 1\\ 2kr\cos\theta \\ 2kr\sin\theta \end{array}\right]$$

where *k* is the signal wavenumber. Considering *L* snapshots, the observation matrix ***X*** is formed by concatenating the array outputs:

(19) X=s⋅a(θ,r)+n

where ***s*** = [*s*(1), *s*(2), …, *s*(*L*)]*^T^* is the complex envelope vector of the signal across *L* snapshots, and ***n*** is the white Gaussian noise matrix, with each element being independent and identically distributed. The CRB is the inverse of the Fisher Information Matrix (FIM). The elements of the FIM are

(20) F=2Re[tr(∑−1∂μ∂θ∑−1∂μH∂θ)]

In the expression, $\sum =\sigma_{n}^{2}I$ are steering vectors, I is the identity matrix, $\sum^{-1}=I/\sigma_{n}^{2}$ and μ=s⋅a(θ,r) is the mean vector, its covariance is $E[\mu \mu^{H}]=\rho_{s}\cdot N\cdot a(\theta ,r)a^{H}(\theta ,r)$. The scalar $\rho_{s}=E[{\left|s\right|}^{2}]$ represent the signal power, *E*[·] represents the statistical expectation of the auto-covariance matrix of the received signal vector, *N* represents the number of array elements. Substituting the defined parameter vector ***μ*** into Equation (20) yields a simplified form.

(21) $$F=2L\cdot SNR\cdot \frac{\partial a^{H}}{\partial \theta }\cdot \frac{\partial a}{\partial \theta }$$

where $SNR=\rho_{S}/\sigma_{n}^{2}$ denotes the SNR. The partial derivative of the steering vector ***a***(*θ*, *r*) with respect to the azimuth *θ* is calculated as

(22) $$\frac{\partial a}{\partial \theta }=\left[\begin{array}{c} 0\\ -2kr\sin\theta \\ 2kr\cos\theta \end{array}\right]$$

Substituting this derivative into Equation (21) yields the FIM:

(23) $$F=2L\cdot SNR\cdot {\left(2kr\right)}^{2}$$

Therefore, the CRB is given by

(24) $$CRB(\theta ,r)=\frac{1}{2L\cdot SNR\cdot {\left(2kr\right)}^{2}}$$

**Figure 9:**
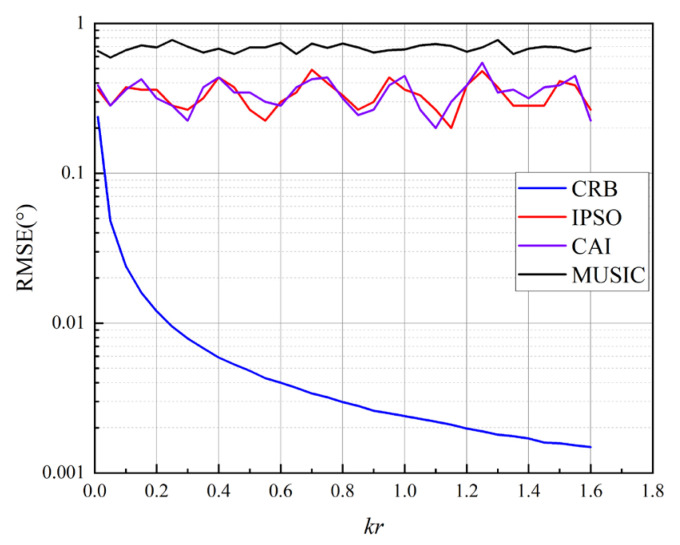
CRB and RMSE versus *kr* for the pressure-gradient MEMS vector hydrophone and its comparison with estimation algorithms.

The CRB for azimuth estimation with a pressure-gradient MEMS vector hydrophone is governed by multiple factors, including the SNR, the source frequency, the number of snapshots, and the hydrophone’s radius. To analyze the characteristics of the CRB across frequency, simulations were configured as follows: hydrophone diameter 2*r* = 0.13 m, SNR = 5 dB, number of snapshots *L* = 7000, and source frequency sweeping from 20 Hz to 3 kHz. Results were averaged over 100 Monte Carlo trials. Figure 9 presents the impact of the normalized wavenumber *kr* on the CRB and on the RMSE of the proposed IPSO, CAI, and MUSIC algorithms. The results indicate that, across the operational bandwidth, the positioning accuracy of the IPSO algorithm is comparable to that of CAI and superior to MUSIC, with both IPSO and CAI performing closer to the CRB. Furthermore, the estimation accuracy of the proposed algorithm remains stable and is essentially independent of the target signal frequency.

### 3.3. Simulation Study

Simulations were performed to analyze the IPSO algorithm from Section 2.2 in comparison with the MUSIC and CAI methods. All results are statistical averages over 100 independent Monte Carlo trials. The simulation environment assumes co-planar target and interference signals propagating in a homogeneous, ideal seawater medium. The system sampling rate was set to 7 kHz with a SNR of 5 dB. A directional interference signal with a center frequency of 450 Hz was placed at 80° azimuth.

To assess the resolution capability of the pressure-gradient MEMS vector hydrophone, two incident target signals were simulated. The first signal had a frequency sweep from 100 Hz to 250 Hz at a fixed azimuth of 60°. The second signal’s frequency ranged from 300 Hz to 450 Hz, while its azimuth was varied at 55°, 50°, 45°, and 40°. This created azimuth separations of 5°, 10°, 15°, and 20° between the two targets, respectively.

**Figure 10:**
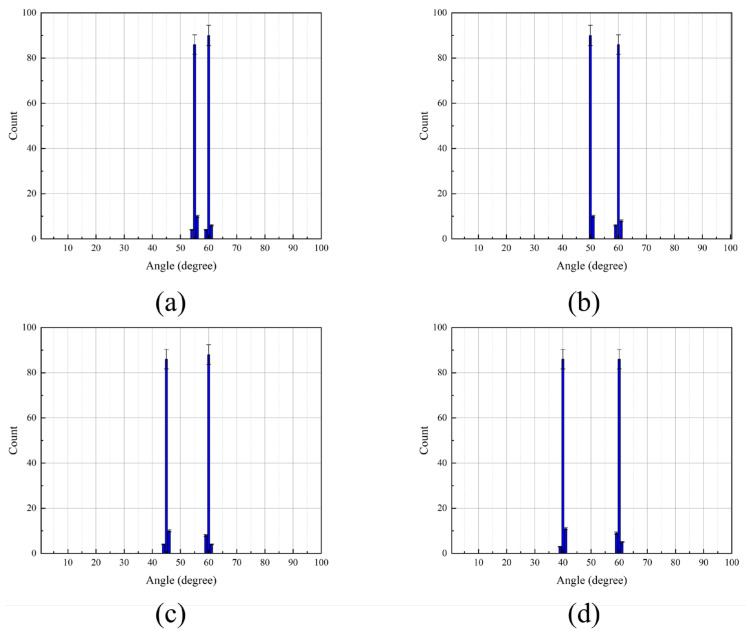
Performance comparison of the IPSO algorithm under different target azimuth separations. (**a**) 5°. (**b**) 10°. (**c**) 15°. (**d**) 20°.

**Figure 11:**
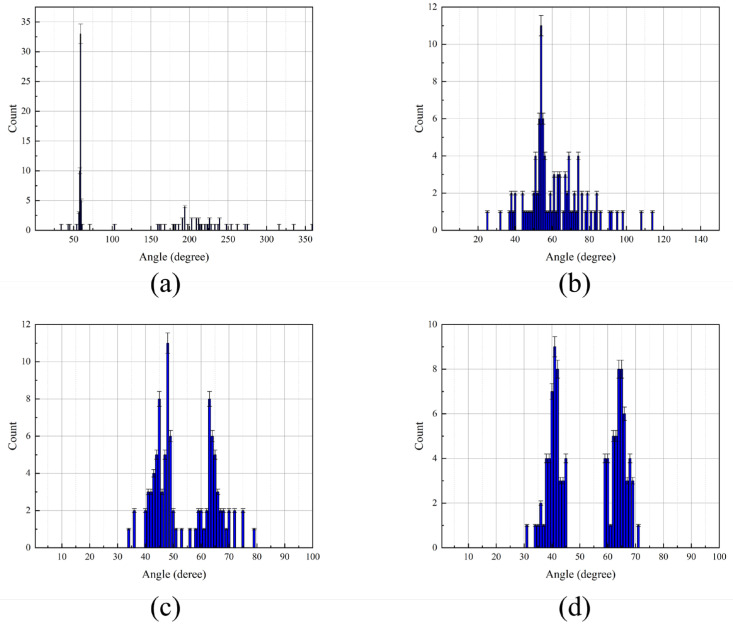
Performance comparison of the MUSIC algorithm under different target azimuth separations. (**a**) 5°. (**b**) 10°. (**c**) 15°. (**d**) 20°.

**Figure 12:**
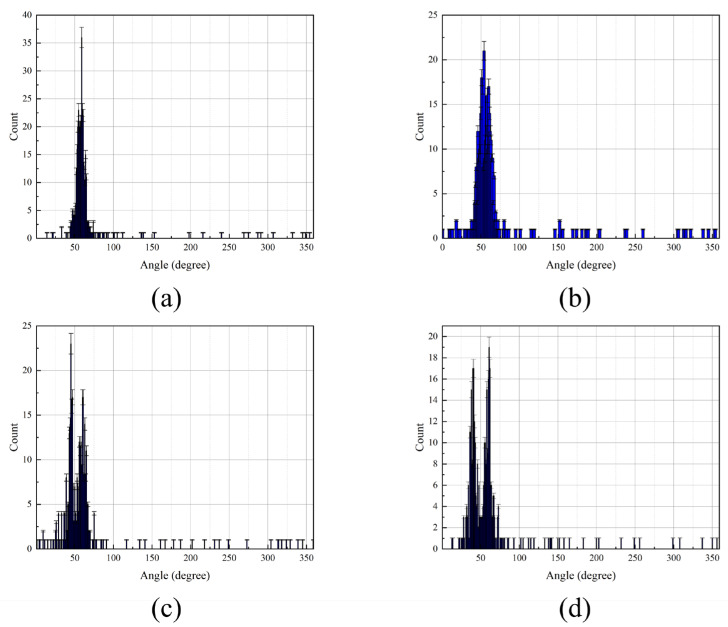
Performance comparison of the CAI algorithm under different target azimuth separations. (**a**) 5°. (**b**) 10°. (**c**) 15°. (**d**) 20°.

As evidenced by Figure 10, Figure 11 and Figure 12 and Table 1, the IPSO algorithm achieves precise azimuth estimation across all tested angular separations. It successfully resolves two closely spaced targets with a RMSE of approximately 0.35° and a 100% probability of successful estimation. These results demonstrate that the IPSO algorithm maintains high-resolution azimuth estimation capability, accurately resolving two targets even at minimal angular separations. Under identical conditions, the MUSIC algorithm suffers from significant performance degradation in low-SNR scenarios, leading to frequent misidentification of target azimuths. In contrast, while the CAI algorithm yields relatively accurate estimates, its probability of successful estimation remains below 90%. Consequently, the IPSO algorithm demonstrates superior overall performance under the challenging conditions of low SNR and small angular separation.

Table 2 presents a computational complexity comparison among the proposed IPSO algorithm, MUSIC, and the CAI method for azimuth estimation using a single pressure-gradient MEMS vector hydrophone. The key parameters are defined as follows: *M* is the number of sensors (here, *M* = 4), *L* is the number of snapshots, *V* is the number of azimuth search points, *S* is the swarm size in IPSO, and *I* is the maximum iteration count. The complexity of all three algorithms is primarily governed by *L* and *V*, leading to differences that are not substantial in asymptotic order. The IPSO algorithm requires iterative updates over *S* particles for *I* generations. However, its computational cost can be effectively managed. This is achieved by judiciously selecting *S* and *I* to balance estimation accuracy with computational load.

### 3.4. Field Validation with Sea Trial Data

**Figure 13:**
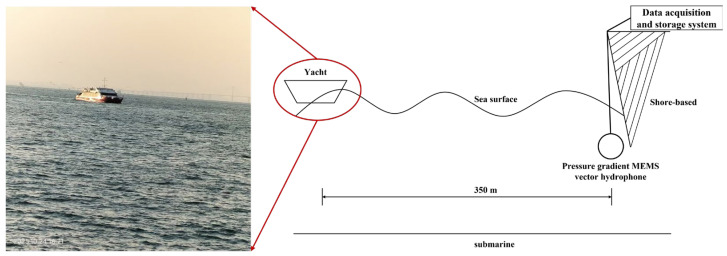
Schematic diagram of the sea trial for the pressure-gradient MEMS vector hydrophone.

To further validate the resolution performance of the IPSO algorithm for a single pressure-gradient MEMS vector hydrophone, a sea trial was conducted. As shown in Figure 13, data were collected in coastal waters near Qingdao using a yacht as an acoustic target. The yacht’s effective radiated noise was concentrated within the 300–800 Hz band, which was used for processing. The data acquisition and storage system was configured with a sampling frequency of 20 kHz and a duration of 200 s per recording. The test site had a water depth of approximately 6 m, with the vector hydrophone deployed at a depth of 4 m and at a horizontal range of about 350 m from the yacht track.

**Figure 14:**
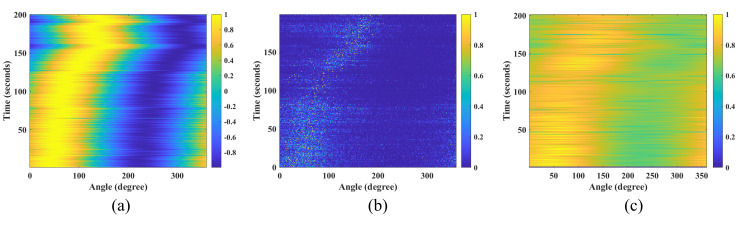
Azimuth estimation results from the IPSO, CAI, and MUSIC algorithms. (**a**) IPSO result. (**b**) CAI result. (**c**) MUSIC result.

**Figure 15:**
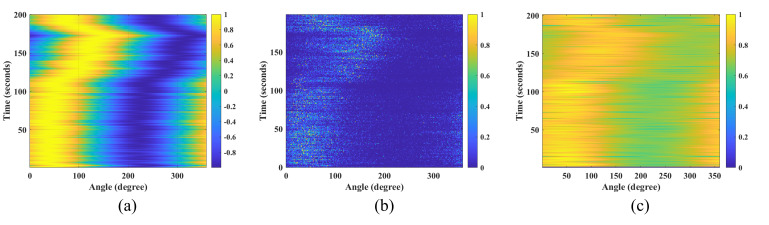
Experimental test of the trajectory diagram for target direction estimation. (**a**) IPSO result. (**b**) CAI result. (**c**) MUSIC result.

A comparison of azimuth estimation performance based on field data is shown in Figure 14 and Figure 15 for the IPSO, MUSIC, and CAI algorithms. Analysis reveals that the CAI method exhibits robustness in estimating the yacht’s azimuth under the test conditions. Conversely, the performance of the MUSIC algorithm is severely compromised by multipath propagation in the ocean environment, leading to substantial inaccuracy. The proposed IPSO algorithm, which incorporates an adaptively tuned inertia weight to regulate the search process, demonstrates superior high-resolution estimation capability. These results confirm the algorithm’s effectiveness and underscore its potential for practical engineering deployment.

## 4. Conclusions

This paper presents an approach for high-resolution azimuth estimation using a single vector hydrophone, which integrates a MEMS acoustic pressure sensor. The core innovation lies in an IPSO algorithm designed for this purpose. The algorithm introduces an adaptively adjusted inertia weight, which effectively resolves azimuth ambiguity and regulates the search process. Comparative simulations demonstrate that the IPSO algorithm achieves superior performance in resolving dual closely spaced targets, confirming its high-resolution capability. Theoretically, we prove the asymptotic unbiasedness of the proposed estimator and derive the corresponding CRB for the pressure-gradient MEMS vector hydrophone, analyzing its dependence on factors such as SNR, signal frequency, snapshot count, and array geometry. Finally, the proposed algorithm is validated using experimental data from a Qingdao nearshore sea trial, demonstrating its high-resolution azimuth estimation capability. Its consistent superiority over MUSIC and CAI methods in these field tests verifies the overall feasibility and practical value of the IPSO algorithm. Nonetheless, the performance of the IPSO algorithm may exhibit a degree of dependence on the initial configuration of its optimization parameters. Furthermore, the generalizability of the proposed model in highly complex multipath scenarios requires further validation.

## Figures and Tables

**Table 1:** Comparison of RMSE and successful resolution probability for the three azimuth estimation algorithms at different angular separations.

Algorithm		5° Separation	10° Separation	15° Separation	20° Separation
IPSO	RMSE	0.35°	0.35°	0.36°	0.37°
	κ	100%	100%	100%	100%
MUSIC	RMSE	76.57°	12.68°	7.19°	4.64°
	κ	8%	9%	3%	21%
CAI	RMSE	1.5°	1.51°	1.38°	1.37°
	κ	79%	84%	88%	89%

**Table 2:** Complexity analysis of IPSO, MUSIC, and CAI algorithms for single-vector-hydrophone azimuth estimation.

Algorithm	The Computational Complexity	Complexity Under Typical Parameters (*L* = 1000)
MUSIC	*O* (*M* ^3^ + *M* ^2^*L* + *M* ^2^*V*)	*O* (21,824)
CAI	*O* (*M* ^2^*L* + *V*)	*O* (16,360)
IPSO	*O* (*M* ^2^*L* + *IS*)	*O* (16,600)

## Data Availability

The raw data supporting the conclusions of this article will be made available by the author on request.
